# Evaluating integrin activation with time-resolved flow cytometry

**DOI:** 10.1117/1.JBO.23.7.075004

**Published:** 2018-07-10

**Authors:** Jesus Sambrano, Alexandre Chigaev, Kapil S. Nichani, Yelena Smagley, Larry A. Sklar, Jessica P. Houston

**Affiliations:** a New Mexico State University, Department of Chemical and Materials Engineering, Las Cruces, New Mexico, United States; b University of New Mexico, School of Medicine and Center for Molecular Discovery, Albuquerque, New Mexico, United States

**Keywords:** flow cytometry, fluorescence lifetime, Förster resonance energy transfer, integrin activation, phasor analysis, protein conformation

## Abstract

Förster resonance energy transfer (FRET) continues to be a useful tool to study movement and interaction between proteins within living cells. When FRET as an optical technique is measured with flow cytometry, conformational changes of proteins can be rapidly measured cell-by-cell for the benefit of screening and profiling. We exploit FRET to study the extent of activation of α4β1 integrin dimers expressed on the surface of leukocytes. The stalk-like transmembrane heterodimers when not active lay bent and upon activation extend outward. Integrin extension is determined by changes in the distance of closest approach between an FRET donor and acceptor, bound at the integrin head and cell membrane, respectively. Time-resolved flow cytometry analysis revealed donor emission increases up to 17%, fluorescence lifetime shifts over 1.0 ns during activation, and FRET efficiencies of 37% and 26% corresponding to the inactive and active integrin state, respectively. Last, a graphical phasor analysis, including population clustering, gating, and formation of an FRET trajectory, added precision to a comparative analysis of populations undergoing FRET, partial donor recovery, and complete donor recovery. This work establishes a quantitative cytometric approach for profiling fluorescence donor decay kinetics during integrin conformational changes on a single-cell level.

## 1 Introduction

Integrins are a family of transmembrane protein heterodimers that are expressed on the surface of different cell types. Very late antigen-4 (VLA-4, α4β1) is one class of integrins expressed on immune cells, such as leukocytes and antigen-presenting cells. These VLA-4 integrins have protein ligands of high affinity (e.g., fibronectin or VCAM-1 for α4 integrin) and function to enable cell-to-cell and cell-to-extracellular matrix interactions, adhesion, and other behaviors important for immune response.^1^^,^^2^ The function and activation of VLA-4 integrins are also dependent on an inside-out cell signaling process mediated by G-protein coupled receptors (GPCRs). Activation of VLA-4 integrins can occur downstream of other immune system-associated receptors. Amid this complexity, the immunological functions that the VLA-4 integrins facilitate are critical; they enable cells to roll and adhere to vasculature as well as promote cell-to-cell and cell-to-pathogen interactions through their ability to modulate ligand-binding affinity.^3^ Such functionality facilitates homing and trafficking of immune cells to specific locations within the human body and is critical to numerous processes that include blood coagulation, cell recruitment to sites of inflammation, pathogen recognition and immune response, learning, memory, fertilization, and cancer cell metastasis.^4^^–^^8^

Interestingly, the functional activity of integrins, and specifically VLA-4, is closely tied to a series of conformational changes that allow the leukocyte cell to adopt diverse modes or behaviors. During different conformational changes, a nonadhesive cell might “float” through blood vessels or in contrast, tightly adhere and crawl throughout lymphoid organs or within sites of inflammation.^9^ Such conformational changes have been observed and corresponding cell behaviors measured in a variety of cell types for multiple integrins. The alteration includes a “switchblade-like” extension of the dimer structure upon activation and lateral separation of the integrin subunits. The movement alters the resting state, in which the hybrid domain conforms by bending to permit the molecule headpiece to lie in close proximity to the cell membrane (i.e., observed on resting cells). The bending occurs at the “knee” or “genu,” in the middle of the integrin molecule between the thigh and the calf-1 domains of the α subunit, and this conformation usually corresponds to the inactive low affinity form. Upon activation, the integrin α and β subunits will laterally separate and the molecule will transition to an extended conformation with the head group and the ligand-binding site pointing away from the cell membrane, thus facilitating an interaction with its ligand. Such conformational changes were reported for the α4β1, αLβ2 integrins and have been validated through exposure of the activation-dependent antibody epitopes and x-ray crystallography to visualize resting and extended integrins.^10^^–^^12^

In prior work and to study this further, we observed integrin conformational changes^13^^–^^17^ on live cells under real-time signaling conditions using a Förster (or fluorescence) resonance energy transfer (FRET) assay.^9^^,^^13^ These early FRET studies were optimized for flow cytometry to determine the average conformational state of groups of activated integrins on the surface of viable cells. A ligand-mimicking donor probe (peptide connected to fluorescein isothyocyanate) was bound to the headgroup of the VLA-4 integrin, and a lipid acceptor, rhodamine, was incorporated into the cell membrane to evaluate integrin molecular extension. FRET was evaluated in an intensity-based assay and interpreted as the modulation of the distance of closest approach between FRET donors and acceptors.^9^ The donor fluorescence intensity was first measured in the absence of the FRET acceptor, representing the unquenched fluorescence of the donor. We then interpreted the inactive resting state of integrin molecules (i.e., bent at the distance of closest approach) by the extent of FRET measured by the decrease in donor emission. As the distance between the donor and acceptor for resting integrins is much less than the Förster distance ($R_{0}\approx 55\;Å$), FRET can be measured. Moreover, subsequent activation of the GPCR-initiated inside-out signaling resulted in rapid integrin extension to a vertical distance ≈200  Å. This extended vertical distance is greater than the effective Förster distance for the fluorescein/rhodamine donor/acceptor pair ($R_{0}\approx 55\;Å$). The effect of the integrin molecules extending past the donor/acceptor Förster distance resulted in a dramatic decrease in energy transfer efficiency. Because the intensity-based measurements were conducted in a conventional flow cytometer, we could not discriminate between different degrees of molecular extension. Morevoer, the fluorescent signal did not recover to the unquenched level, which led to the interpretation of only a fraction of integrins being partially extended.^9^ Therefore, in this study, we take donor fluorescence lifetime measurements to add to the interpretation of integrin extension while retaining the throughput necessary for a flow cytometry assay. We thus provide a first look into the heterogeneity of integrin responses in a cell population.

Fluorescence decay kinetic measurements are valuable for FRET studies and add quantifiable information in a calibration-free way. In fact, others^18^ have used FRET to identify effectors that elicit integrin activation and inhibitors that act as integrin antagonists. Such studies use fluorescence lifetime imaging microscopy (FLIM) on VLA-4 integrins and LFA-1 (αLβ2), where FLIM is used to evaluate FRET between a green fluorescence protein donor and red fluorescence protein acceptor. FRET was measured with FLIM for the donor when expressed with the integrin and acceptor when bound to effectors talin, α-actinin, and paxillin. This approach proved that β2 integrin interacts with talin and α-actinin effectors. Additionally, a significant loss of FRET was observed due in part to the decrease in α4 integrin binding with paxillin. Conformational changes were also observed^19^ with fibronectin receptors α5β1, in which the heterodimeric integrins were forced into a resting state in the presence of an agonist. Regions of cells where integrin activation was most prevalent were identified by FLIM-FRET. This was also taken a step further by conjugating fragment antigen-binding (Fab) to Alexa Fluor 546. The Fab-Alexa546 donor molecule targeted a fibronectrin receptor. Therefore with an acceptor anti-integrin monoclonal antibody (mAb)-bound VC5, at the cell membrane, loss of FRET was an indication of activation (i.e., absence of the acceptor, donor lifetime=2.6±0.2  ns, presence of the acceptor, and donor lifetime=1.8±0.2  ns).

The use of fluorescence decay kinetic measurements is known for adding a quantitative value to FRET because the fluorescence lifetime is independent of fluorophore (donor probe, LDV-FITC) concentration. Despite the amount of donor present, when not interacting with an acceptor molecule, the donor fluorescence lifetime is constant, whereas fluorescence intensity measurements are dependent on the donor probe concentration (higher concentration equals brighter intensity). However, it is noteworthy that donor concentration is certainly consequential at higher concentrations as fluorophore–fluorophore (donor–donor) interaction becomes more probable. Since the fluorescence lifetime of the donor molecule shortens during FRET, this hallmark of nonradiative energy transfer is a straight-forward indicator of FRET. In this contribution, we use a digital frequency-domain flow cytometer^20^^,^^21^ to develop a phasor-based prediction of FRET and calculate the fluorescence lifetimes of FRET donor molecules during integrin activation. The idea that decay kinetic measurements lead to direct evaluation of FRET efficiency with less experimental controls or extensive calibration and normalization procedures is shown. The approach includes simple frequency-domain hardware modifications and signal processing techniques to report physiological transformation of integrins when activated.

## 2 Theory

**Fig. 1:**
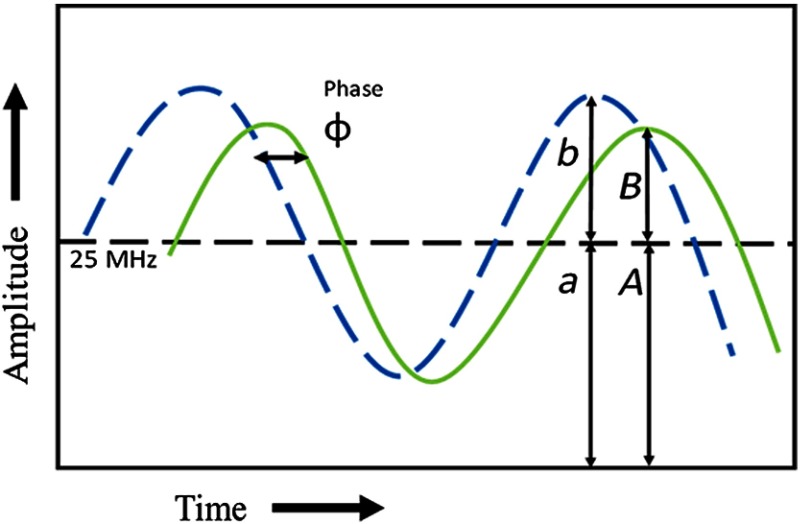
A theoretical sinusoidal, frequency-domain signal depicted in one cycle. An excitation source (e.g., diode laser) is modulated at a specified frequency (blue, dashed line). The collected green fluorescence (green, solid line) is time delayed, denoted by ϕ, and demodulated, where modulation depths are defined as $m_{\mathrm{ex}}=b/a$ for the excitation signal and $m_{\mathrm{em}}=B/A$ for the emission signal.

Time-resolved flow cytometers measure the fluorescence lifetime of fluorophores in or on the surface of cells as the cells move through the path of a finely focused laser beam.^21^ The throughput of cell counting (near 1000  cells/s) is retained while decay-dependent values (e.g., phase lifetime, modulation lifetime, real and imaginary component of resulting frequency space, etc.)^22^^–^^24^ are computed for sorting and/or analysis. We have developed a series of different iterations of time-resolved flow cytometry (TRFC), mainly involving frequency-domain approaches^20^^,^^22^^,^^23^^,^^25^ by choosing a laser modulation frequency and performing homodyning during cell passage through the laser. With this approach, the time of fluorescence decay (nanoseconds) can be calculated by tracking the emission that is phase shifted and amplitude attenuated relative to the excitation light. The frequency-domain concept is shown in Fig. 1. Demonstrations of frequency-domain flow cytometry mainly differ by how the emission signals are processed, which generally involve either analog or digital approaches fostered by on-chip Fourier analyses.^20^^,^^26^^,^^27^ Use of digital signal processing permits the acquisition of the measured decay in real time given certain assumptions about the exponential decay. When a single-exponential decay is assumed, the average fluorescence lifetime can be calculated quickly and used for cell sorting.^20^^,^^26^^,^^27^ Moreover, the accuracy of the fluorescence lifetime can range depending on the instrumentation and time-resolved approach taken. For example, others have constructed microfluidic-based time-domain cytometers that have increased resolution and sensitivity at the expense of speed.^28^

Briefly with frequency-domain flow cytometry, the average fluorescence lifetime, τ, is taken to be proportional to the phase shift of a frequency-modulated fluorescence signal. The mathematical representation of this concept is provided by

$$\tau_{\phi }=\frac{\tan\;\phi }{\omega },$$ (1)

where ϕ is the phase shift between the excitation phase and the emission phase, ω is the angular frequency, and $\tau_{\phi }$ is the phase lifetime. Similarly, the amplitude demodulation of a frequency-modulated fluorescence signal can be used to calculate the fluorescence lifetime, as shown below. For the relationships

$$m_{\omega }=\frac{1}{1+\omega^{2}\tau^{2}},\;\mathrm{and}\;\tau_{\omega }=\frac{\sqrt{{\left(\frac{m_{\mathrm{ex}}}{m_{\mathrm{em}}}\right)}^{2}-1}}{\omega },$$ (2)

where m is equal to the demodulation of the emission signal, ω is the angular frequency, $\tau_{\omega }$ is the modulation lifetime, $m_{\mathrm{ex}}$ is the modulation of the excitation signal, and $m_{\mathrm{em}}$ is the modulation of the emission signal.^29^

The fluorescence signals acquired with our time-resolved cytometer are frequency modulated, which allows us to perform Fourier analyses to extract the phase shift between the fluorescence signal and the excitation signal (taken as the side-scattered signal) and amplitude demodulation, which is the ratio of emission modulation depth with respect to the excitation modulation depth. Furthermore, concurrent signal processing of the complex Fourier output is implemented to construct a polar plot representation of the phase shift and amplitude demodulation. The polar, or “phasor” plot,^30^ graphically represents the frequency-domain signals as vectors where the angle is equivalent to the phase shift, and the magnitude represents the demodulation amplitude. Phasor graphs, which are common displays of FLIM data, permit the visualization of two lifetime-dependent parameters (phase and modulation) on a single dimension or plane plot.^30^^–^^35^ Ideally, these plots are useful to visualize measurements of multiple fluorescence decay kinetic events (i.e., different fluorescence lifetimes). Moreover, they are useful in identifying and visualizing the cell populations undergoing resonance energy transfer.^32^^,^^36^^,^^37^

In past work, we have implemented pseudo-phasor and full phasor displays with the intent to develop a cell-by-cell visualization tool for the multiple fluorescence lifetimes expressed within each cell.^24^^,^^38^ Building on this work, herein, we generate an FRET “dequenching path” using the experimental data points on the phasor plot. It is well established that phasors for mixture components or multiple lifetime components can be decomposed into constituent species and fluorescence lifetimes using linear interpolation.^38^ Yet with FRET data, unmixing using linear combination is not possible. Instead, tracing a curved path along the different cell populations undergoing loss of FRET enables us to extract and evaluate FRET efficiencies. The FRET efficiencies for each cell population can be calculated using fluorescence lifetime measurements of the donor molecule as provided by the following:

$$\mathrm{FRET}\text{ efficiency}(E)=1-\frac{\tau_{\mathrm{DA}}}{\tau_{D}},$$ (3)

where $\tau_{\mathrm{DA}}$ is the fluorescence lifetime of the donor in presence of acceptor and $\tau_{D}$ is the lifetime of the donor only. Using the lifetime information from the donor channel from our FRET protocol, the dequenching path is constructed using a generalized cubic spline (Catmull–Rom) interpolation.^39^ Assuming FRET efficiencies calculated at various points of the dequenching path directly translates to the extent of integrin activation on the cell surface. The dequenching path can be used for quantitative estimation of integrin activation. Furthermore, distribution of cell populations in the phasor space permits segmentation into meaningful clustered cell populations. Such segmentation can be performed by manual gating processes or use of automatic clustering algorithms. The clustering of the cell populations was performed using a density-based algorithm implemented in an R programming package called SamSPECTRAL.^40^ Parsed function parameters were adjusted to obtain a desired number of visually meaningful ensembles of cellular events.

## 3 Materials and Methods

### 3.1 Cells

A human monoblastoid cell line, U937, was used for the integrin modulation study (ATCC^®^ Manassas, Virginia). The U937 cells, stably transfected with a nondesensitizing mutant of the formyl peptide receptor (FPR1 ΔST),^41^ were a gift from Dr. Prossnitz.^42^ Cells were cultured in RPMI 1640 GlutaMAX media containing 25 mM HEPES, ${\mathrm{Ca}}^{2+}$ (0.40 mM), and ${\mathrm{Mg}}^{2+}$ (0.42 mM) cations, as well as 10% fetal bovine serum and 1:100 dilution of 100× penicillin, streptomycin, and l-glutamine (Life Technologies, Staten Island, New York). Incubation periods for culture conditions averaged 37°C at 5% ${\mathrm{CO}}_{2}$ concentration with a mean relative humidity of 81%.

### 3.2 Cell-Specific FRET Probes

The FRET donor and acceptor molecules were developed as follows. A peptide derivative probe based on a high affinity VLA-4 specific ligand, 4-[(N-2-methylphenyl)ureido]-phenylacetyl-l-leucyl-l–aspatyl-l-valyl-l-prolyl-l-alanyl-l-lysine (LDV peptide), was conjugated to fluorescein isothiocyanate (LDV-FITC, American International Biotechnology, LLC., Richmond, Virginia). The FRET donor is abbreviated as: LDV-FITC.^17^ The FRET acceptor molecule was a red lipophilic fluorescent dye, PKH26 (Sigma-Aldrich, St. Louis, Missouri), which preferentially accumulates within the cellular membrane. In prior work,^9^ the acceptor fluorophore was found to immediately quench LDV-FITC fluorescence when in close proximity; thus, the addition of the fluorescence lifetime as an added parameter leads to direct quantification of FRET efficiency and thus interpretation of integrin conformational changes without the need for experimental controls, calibration, and normalization.

### 3.3 Experimental Setup of the FRET Protocol

**Fig. 2:**
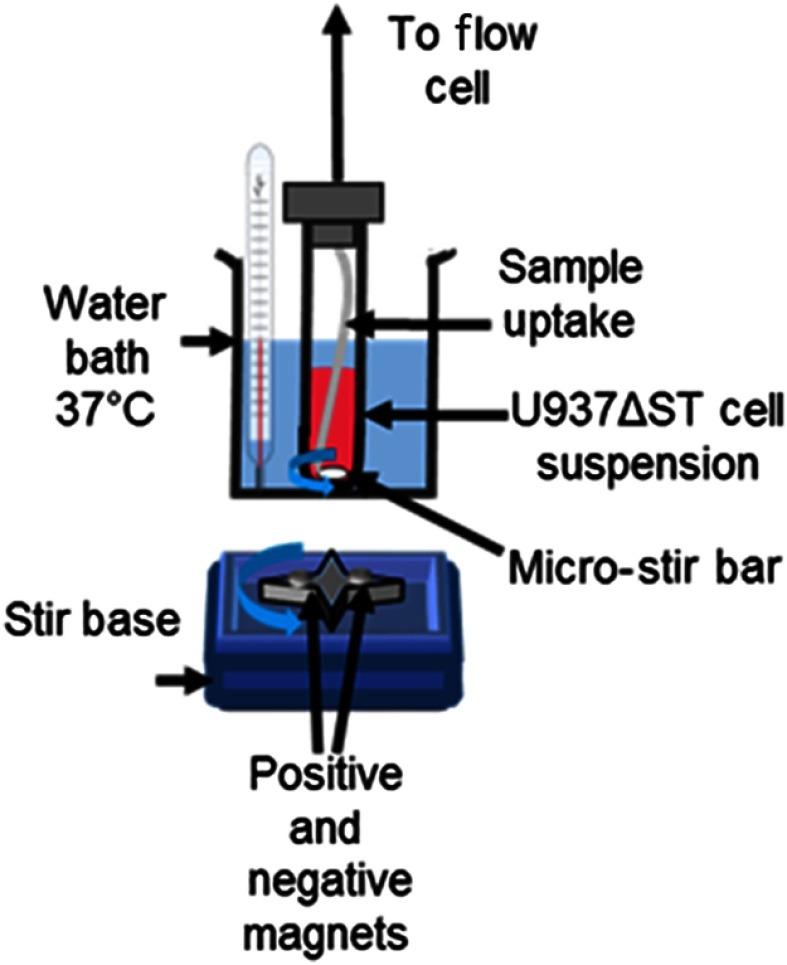
Apparatus used to maintain cell viability during FRET experiment. A water bath is maintained at 37°C while concurrent stirring is applied by a retrofitted stir system (computer fan with attached polarizing magnets and microstir bar). Addition of fluorophores and artificial activator requires sample tube to be taken out of the apparatus with negligible effect on measurements. The sample is drawn through the uptake nozzle and channel to the flow cell for analysis.

Cell suspensions (U937) were transfected with a nondesensitizing mutant of the formyl peptide receptor (FPR1 ΔST).^41^ This mutant has been shown to signal over a longer duration than the wild-type receptor, and therefore, the activated VLA-4 conformation was sustained for up to 1000 s.^17^ Cells were continuously stirred but not vigorously during the flow cytometry measurements in order to maintain uniform probe and ligand distribution. Stirring also enhances natural cellular circulation and migrating motion. Microstir bars were used; bars were placed inside tubes containing the cell suspensions, which were also maintained in a water bath at 37°C. This setup is shown in Fig. 2. Cells were momentarily taken off the bath and stirring was paused during the addition of LDV-FITC (100 nM), PKH26 (2  μM), and fMLFF (100 nM), respectively. “Down” times did not exceed more than 15 s; thus, there were negligible effects on intensity and time-resolved measurements.

**Fig. 3:**
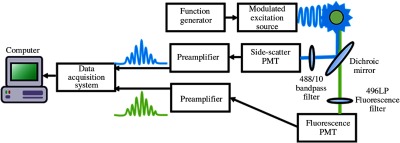
An overview of a TRFC. Modulated laser light intersects and excites fluorescently labeled cells traversing through a fluidic chamber (green circle). A focusing lens, optical filters [488/10-nm bandpass filter for side scatter and 496-nm long-pass (LP) filter for fluorescence], and dichroic mirrors are used to collect side-scattered light (blue trace) and fluorescence (green trace) using PMT detectors. Both side-scatter and fluorescence signals are amplified (“preamplifiers”) and sent to a data acquisition system for digitization at high-sampling rates. Data are displayed in real time (via computer).

Flow cytometry FRET data were taken using two cytometry systems, a standard intensity-based flow cytometer and a time-resolved flow cytometer (Fig. 3). The fluorescence intensity measurements were acquired with a BD Accuri™ C6 Cytometer (BD Biosciences, Franklin Park, New Jersey). Time-resolved measurements were acquired with a modified BD FACSVantage SE (BD Biosciences, Franklin Park, New Jersey). This cell-sorting cytometer utilizes the native fluidic lines and related cytometric components. A Coherent Inc., OBIS 488-nm (Santa Clara, California), 50-mW diode laser was used for our experiment. Digital sinusoidal modulation of 25 MHz was applied to the laser with an arbitrary function generator (Tektronik AFG-3102, Beaverton, Oregon). Photodetection was made possible with photomultiplier tube (PMT) detectors (R1477-04, Hamamatsu Photonics, Lake Forest, California). Signals were amplified with preamplifiers (60 dB, AC-100, Advanced Research Instruments Corp., Bandon, Oregon) and digitized at 250 mega samples per second through a field-programmable gate array by an X5-210M (Innovative Integration, Simi Valley, California) logic board in a custom data acquisition system (DarklingX, LLC, Los Alamos, New Mexico). Prior to collection of cytometry data, laser alignment was optimized as were voltage settings on the photodetectors for the range in emission signals anticipated. The BD Accuri™ C6 Cytometer did not require calibration, and the FACSVantage™ was aligned using CellQuest™ Pro software (San Jose, California). Additionally, the time-resolved measurements required calibration with fluorescence samples of known (*a priori*) fluorescence lifetimes. For this calibration, Flow-Check™ Fluorospheres (Beckman Coulter, California) were used, which have reported average fluorescence lifetime of 7 ns.^20^^,^^26^

### 3.4 Initiating Integrin Conformational Changes and FRET

**Fig. 4:**
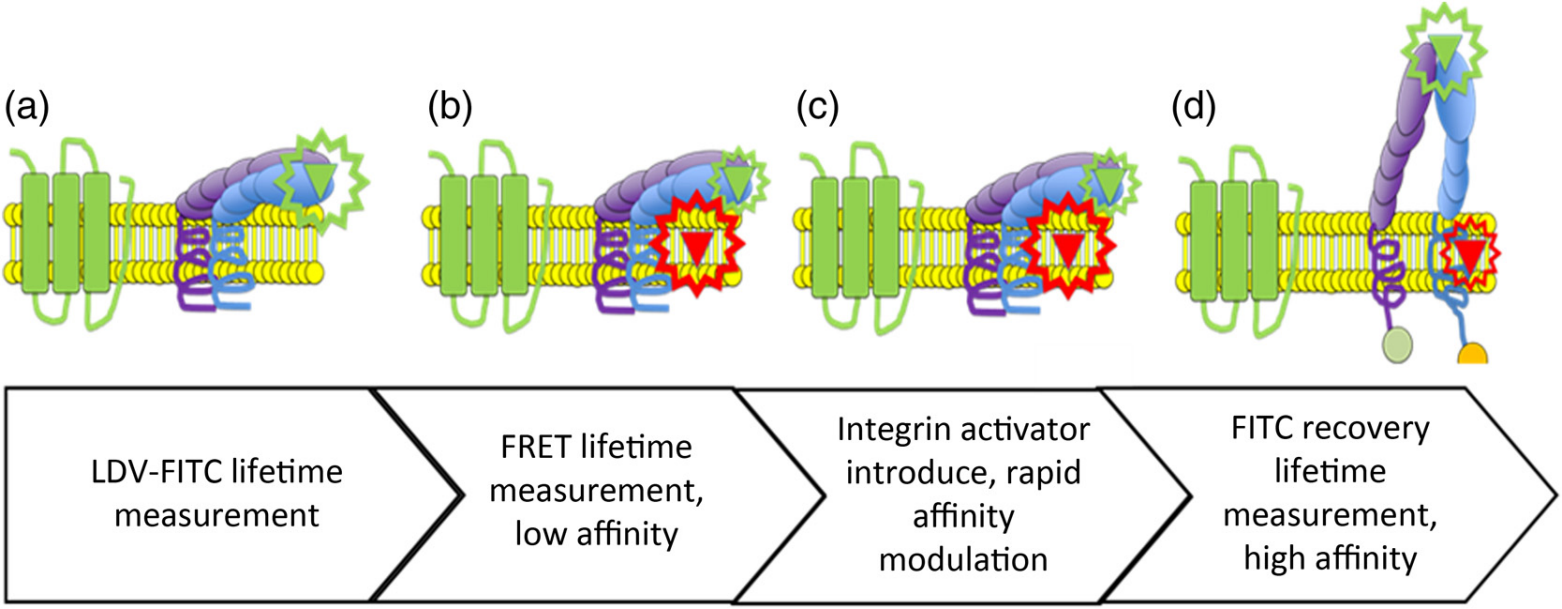
Stage-by-stage depiction of the integrin conformational changes. The U937 cells transfected with a nondesensitizing mutant of the formyl peptide receptor (FPR1 ΔST), constitutively expressing target integrin dimer α4β1, were studied in their: (a) low affinity, resting (bent) conformation. Here, the donor probe (LDV-FITC, 100 nM) was introduced into the cell suspension. This resulted in the rapid binding of the probe to its specific binding site on the α4 integrin headgroup. As reported previously, 100-nM LDV-FITC concentration was sufficient to fully saturate all low affinity VLA-4 sites and the subsequent inside-out activation did not cause any additional probe binding. (b) The acceptor probe PKH26 is introduced into the cell suspension, where it rapidly partitioned into the plasma membrane. As shown previously, this results in the rapid quenching of the donor fluorescence. (c) The GPCR-induced inside-out integrin activation. The high affinity ligand for FPR1, N-formyl-Met-Leu-Phe-Phe-OH peptide (fMLFF) was added at a saturating concentration. This triggered a series of intracellular signaling events that led to modulation of the VLA-4 integrin ligand-binding affinity and promoted the extended integrin conformation. An increase in the distance of closest approach between FRET donor and acceptor results in the loss of FRET and rapid dequenching of donor emission. (d) The loss of FRET is observed.

The procedural steps taken to initiate FRET and loss of FRET involve sequential labeling of cell surface integrins with the donor fluorophores and the cell membrane with acceptor fluorophores. The FRET study presented in this contribution is visualized in Fig. 4. A cell suspension of $1\times 10^{6}\text{ cells}/\mathrm{mL}$ is used in each trial of the experiment and while contained inside the previously discussed apparatus setup. The following FRET protocol applies to fluorescence intensities and time-resolved measurements: (1) donor fluorophore LDV-FITC with a known affinity for the VLA-4 integrin is used to label cells at a concentration of 100 nM; (2) after LDV-FITC addition, ≈10,000 cells are counted and measured; (3) 2  μM of PKH26 acceptor is then introduced into the same cell population, and cell counting is continued for another 10,000 events (at this stage, the addition of the acceptor fluorophore quenches the LDV-FITC donor); and (4) the high affinity FPR1-specific ligand and integrin activator (N-formyl-Met-Leu-Phe-Phe, fMLFF, Sigma-Aldrich, St. Louis, Missouri) at 100 nM is added to the same population to activate integrins into an extended state causing a loss of FRET.^41^^,^^42^ Subsequent to fMLFF addition, another 10,000 cells are counted by cytometry.

## 4 Results

### 4.1 Conventional Flow Cytometry

**Fig. 5:**
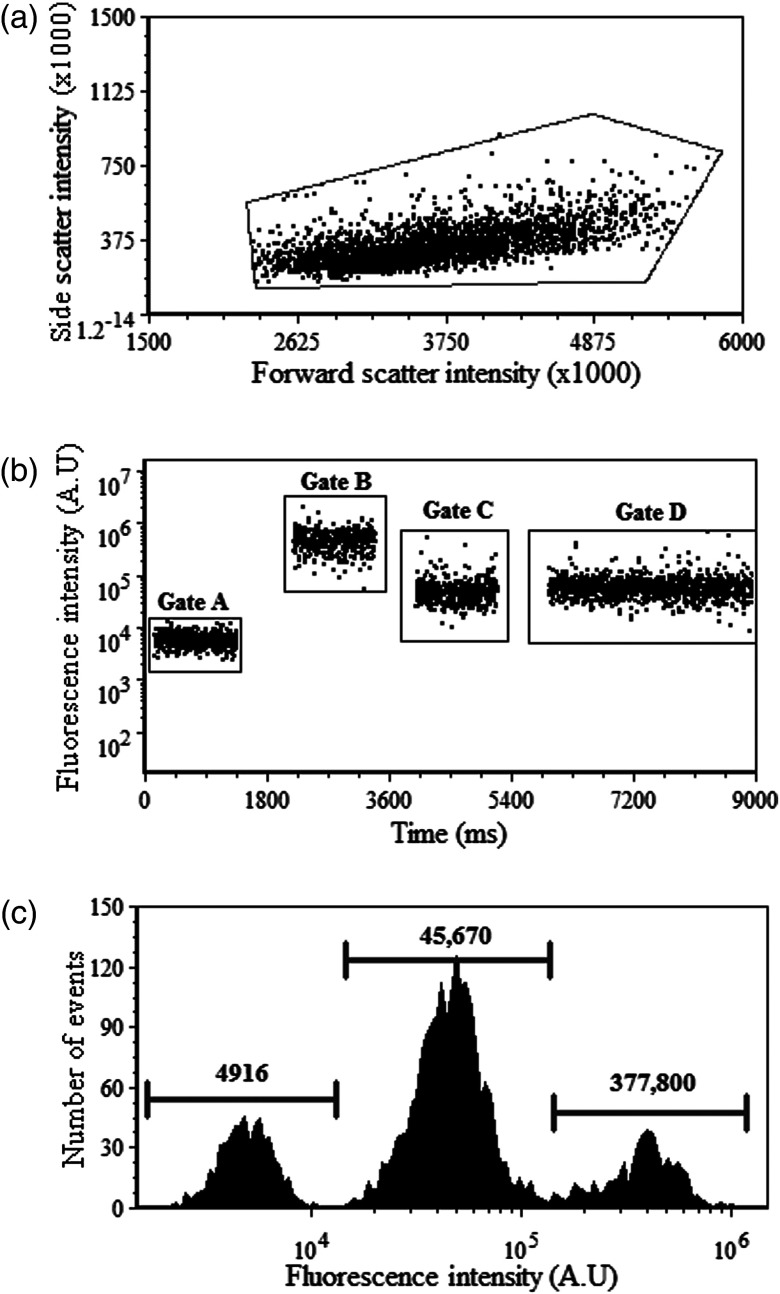
Flow cytometry evaluation of FRET. (a) Dot plot of forward-scatter versus side-scatter light, indicating gated region of cells and (b) fluorescence intensity versus time to depict changes that correlate when cells are labeled and treated for FRET and loss of FRET. Cell populations are time gated to rectify outliers. Gate $A:{\mathrm{MFI}}_{\text{autofluorescence}}=4881$ and CV=28%, gate $B:{\mathrm{MFI}}_{\mathrm{LDV}\text{-}\mathrm{FITC}}=379,900$ and CV=42%, gate $C:{\mathrm{MFI}}_{\mathrm{FRET}}=41,660$ with a CV=55%, and gate $D:{\mathrm{MFI}}_{\text{Loss of }\mathrm{FRET}}=48,250$ with CV=56%. (c) Overlay of histograms of autofluorescence intensities acquired pre-FRET, fluorescence intensity of donor probe before FRET, during FRET, and donor dequenching (integrin activation).

Integrin conformational changes were first quantified with fluorescence intensity measurements using conventional flow cytometry. Gating was applied to eliminate cellular debris by observing side scatter and fluorescence intensity [see Fig. 5(a)]. For each repeated experiment, mean fluorescence intensities (MFI) were collected in triplicate and averaged per experiment. Time versus fluorescence intensity was plotted to visualize the changes in emission with regard to changes in integrin orientation, shown in Fig. 5(b). The full range of emission signal collected in this channel includes a starting point where unlabeled cells (i.e., autofluorescence) are first measured, followed by fluorescence measured before, during, and after FRET. All values are plotted versus time and in cytometric histogram format [Fig. 5(c)]. Autofluorescence from unlabeled cells had an MFI equivalent to 4880 arbitrary intensity units (A.U.) with a CV=28%. Whereas the LDV-FITC donor probe in the absence of the acceptor emitted with an MFI equivalent to 379,900 A.U. and CV=42%. Addition of acceptor probe PKH26 subsequently decreased this mean intensity to 41,660 A.U. with a CV=55%. To cause restoration of LDV-FITC fluorescence, integrins in a bent state are erected by the artificial activator, fMLFF, and intensities were monitored for 1000 s. The MFI increased to 48,250 A.U. with a respective CV=56%. It is important to note the measured intensity discrepancies in Figs. 5(b) and 5(c). An additional time gate is applied to the cell population gate in Fig. 5(b) to further filter outlier events. Figure 5(c) measured values are based on the applied population gate seen in Fig. 5(a).

### 4.2 Time-Resolved Measurements for Average Fluorescence Lifetime

**Fig. 6:**
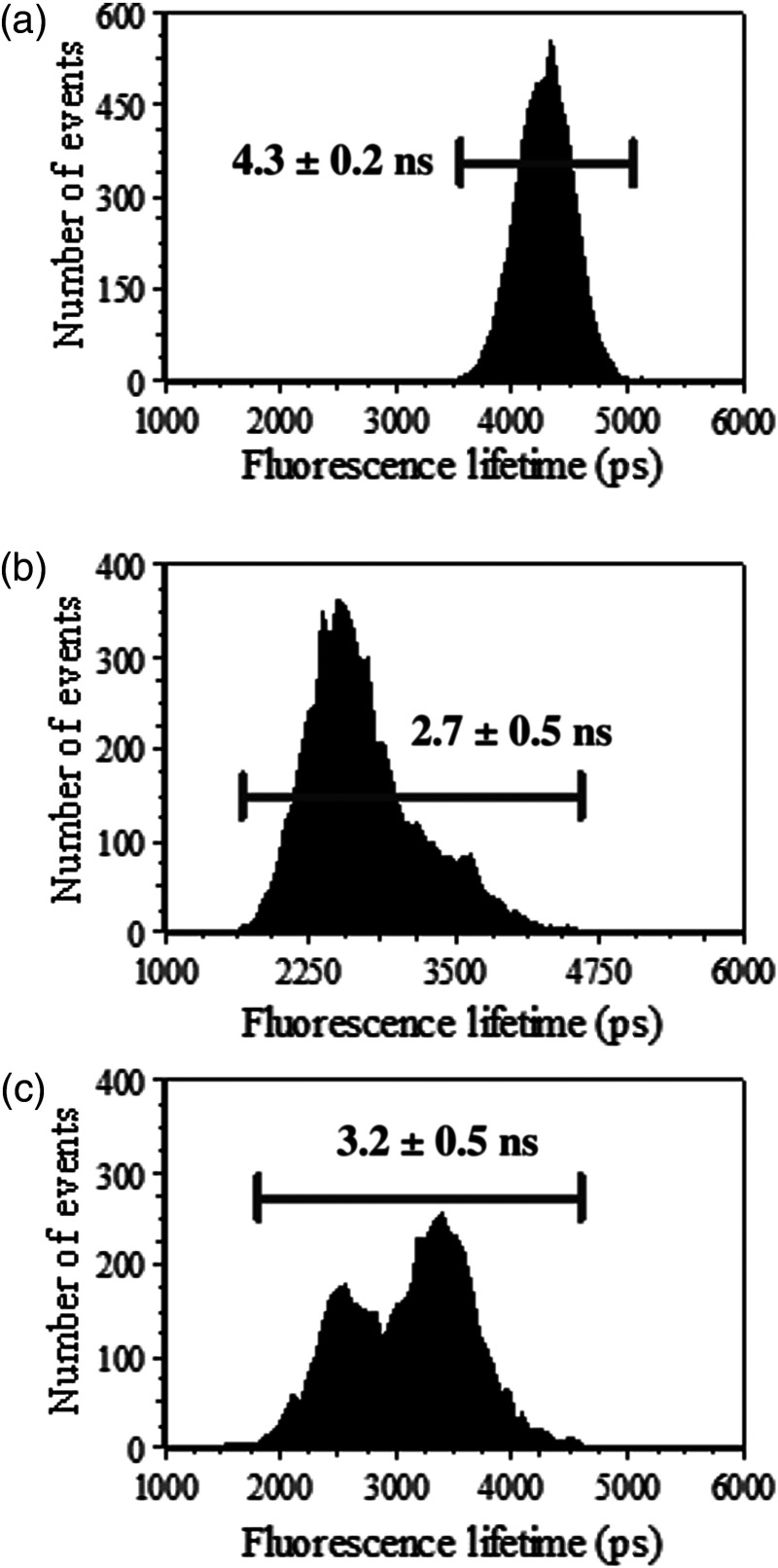
Average fluorescence lifetime values measured per integrin conformational event. Fluorescence lifetime values were obtained for LDV-FITC donor probe, quenching of the donor probe, and dequenching of the donor probe. (a) The average LDV-FITC donor lifetime=4.3±0.2  ns, (b) the average FRET lifetime=2.7±0.5  ns, and (c) average loss of FRET lifetime=3.2±0.5  ns.

Similar to the FRET protocol performed to acquire fluorescence intensities, time-resolved measurements were acquired in triplicate on a per experiment basis as seen in Fig. 6. On average, 15,000 events were recorded per experiment and gating was applied to filter out outliers and cellular debris. The mean fluorescence lifetime for LDV-FITC fluorescently labeled cells was recorded at 4.3 ns with a standard deviation of ±0.2  ns. During energy transfer, LDV-FITC resulted in a noticeably different fluorescence lifetime, this being 2.7  ns±0.5  ns. Activation of integrins (LDV-FITC dequenched) resulted in an LDV-FITC fluorescence lifetime increase to 3.2  ns±0.5  ns. As expected, as LDV-FITC fluorescence lifetime recovery follows expected movement of the α4β1 integrins away from the cell membrane labeled with PKH26 into a nonquenching region. These data show that the LDV-FITC fluorescence lifetime increased but not to levels measured of the donor in the absence of the acceptor. Additionally, the acquired fluorescence lifetime histograms broaden during FRET and integrin activation owing to the presence of subpopulations of integrins with no or minimal activation.

### 4.3 Phasor-Based Calculations and FRET Efficiency

**Fig. 7:**
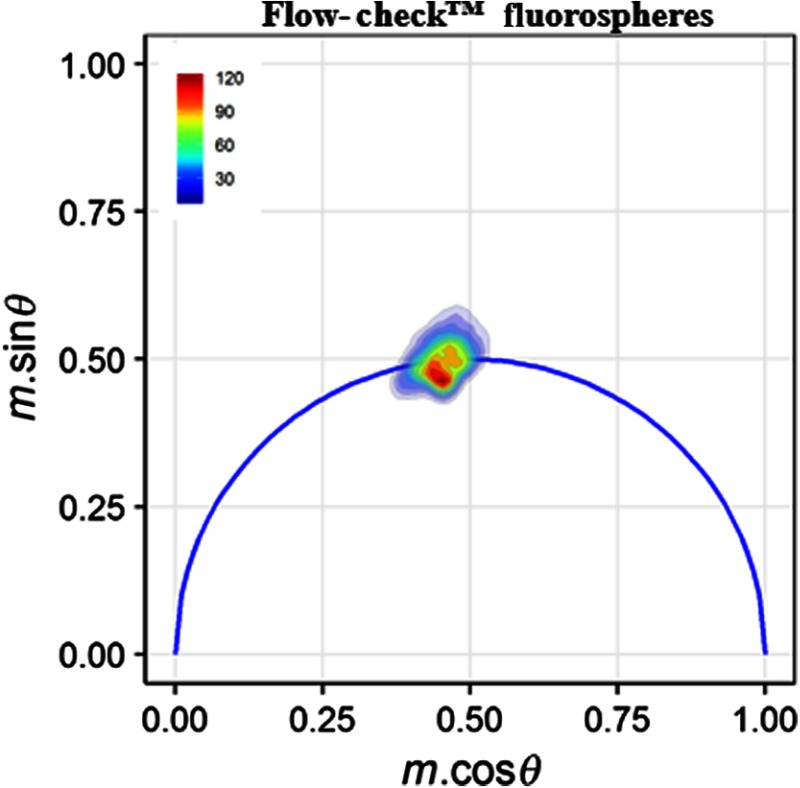
A phasor plot for Flow-Check™ fluorospheres (Beckman-Coulter) is used for calibration purposes at 25 MHz. Polar (phasor) plots are generated in MATLAB for each event to examine the distribution of fluorescence lifetimes and drawn using graphical packages in R (r-project.org). A single clustered population is illustrated on a semicircle, indicative of single-exponential lifetime.

Further time-resolved analyses include the development of phasor graphs. The process involves taking a fast Fourier transform on the data to transpose the signals into frequency spectra for analysis of amplitude and phase. Then, a phasor transposes the polar values to the Cartesian values, where the y-axis is the modulation values times the cosine of the phase and x-axis is the modulation values times the sine of the phase value. A semicircular boundary represents the outer edges for a single fluorescence lifetime phase and modulation. When a single cell’s resultant value falls inside the semicircle, it is indicative of fluorescence kinetics that decay with greater than one exponential component. Results of our calibration with Flow-Check™ fluorospheres resulted in a tight, clustered population as shown in Fig. 7. The distribution is a result of the measurement of digitized signals from the fluorospheres using fluorescence and side scatter to calculate the demodulation and phase shift values. As seen in this phasor, the total population hovers over the universal phasor circle where some values are in the circle, on the line, or outside the circle. The variance is owing to the stochastic nature of the fluorescence lifetimes as well as how high the digitization of the frequency-domain signal is achievable. All graphed results are performed in R (r-project.org). Once calibrated, phasor graphs were developed for the following events: LDV-FITC in the absence of PKH26, quenched LDV-FITC, and donor fluorescence recovery. For all phasor graphs, each “dot” represents a cell/fluorescent fluorosphere, and the populations are displayed as density regions. Also important to note in these results is the fact that the single-exponential nature of LDV-FITC in this study is unlike the single-exponential nature of the fluorescent fluorospheres (i.e., uniformity differs because cells have wider ranging sizes, shapes, and degrees of fluorescent labeling).

**Fig. 8:**
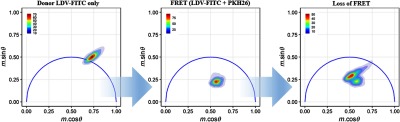
Graphical representation of the FRET experiment in phasor plots. After calibration, the demodulation depth and phase values are plotted on phasor graphs. Plots follow order in which the analysis was performed; donor fluorescence lifetime, showing a consensus for a single lifetime value. FRET (FITC donor quenched by acceptor PKH26) with the population moving inside the semicircle indicative of multiple lifetime values and loss of FRET with bimodal populations present, showing the nonresponsive population, partial recovery population, and recovered population.

The phasor representation of the full FRET experiment is shown in Fig. 8. The populations that progressively change depending on the decay kinetics measured are depicted. First, the native donor with no other fluorophore or activator added shows a population falling on the outer semicircle indicating one dominating fluorescence decay time, as expected. The tightly clustered population then migrates inside the semicircle as the donor undergoes FRET when the acceptor molecule is intercalated into the cell membrane. Finally, the activator is introduced to activate the integrins and the population shifts to a higher phase angle with a lower demodulation depth (i.e., fluorescence brightness during recovery is less than FITC alone). Event positions on the phasor plot depict when populations reach full recovery or have decay kinetic shifts that differ or distribute across a specific multiexponential region, permitting clustering into different populations.

**Fig. 9:**
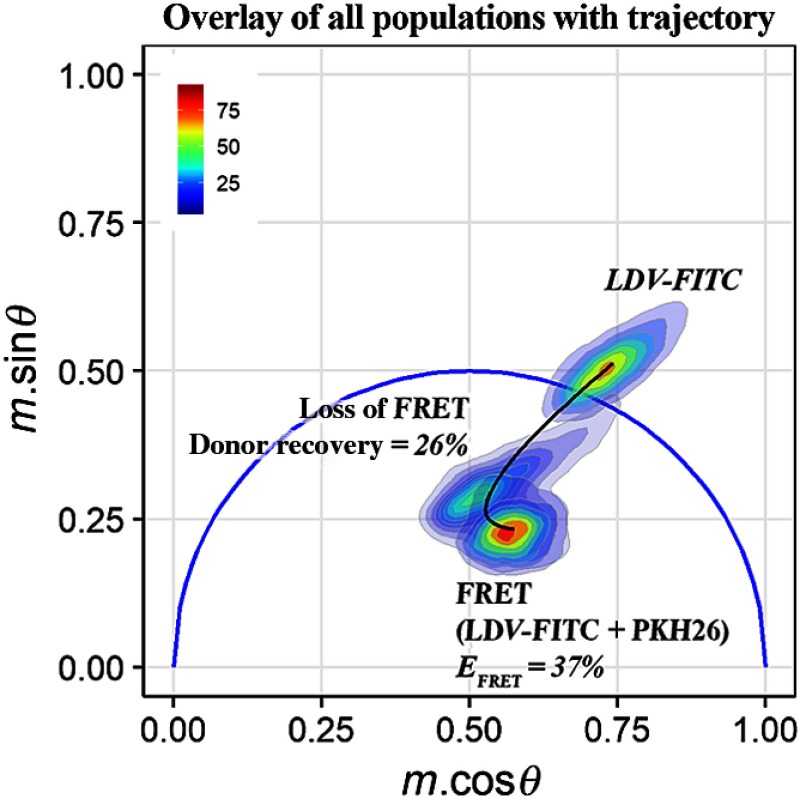
Phasor overlay showing LDV-FITC, FRET, and loss of FRET population distributions with trajectory. Trajectory tracks the donor’s decay kinetics as it undergoes FRET, followed by its recovery. The trajectory allows evaluation of FRET efficiencies by observing the order of events in the phasor space. The FRET efficiency was calculated to be 37%. In addition, the donor recovery post-FRET was calculated at 26%. This plot is substantially quantitative as it adds a spatial component to compliment the temporal FRET evaluation.

Figure 9 combines the FRET analyses together by representing the nonlinear combination of lifetimes. When combined, an FRET trajectory^32^^,^^37^ can be traced. A cytometric FRET trajectory differs from phasor-based trajectories made in microscopy, which contain pixel data from the donor and acceptor channels. With cytometry, the trace (Fig. 9) represents a complex temporal profile for the entire cell population during a representative experiment as cells move from a quenched to a dequenched state. A path is predicted by finding the median phasor values of the cell populations in each stage, which correspond to the changing photophysical states of cell-bound FITC. The median value for the cell population corresponding to loss of FRET is obtained after segmenting into two clusters: one clustered cell population containing events undergoing FRET and the second clustered cell population corresponding to cells in various stages of donor recovery. Thus, the path is characteristic of the process of integrin activation. Since we are generally interested in the extended open confirmation of the integrins for therapeutic development (due to increased ligand affinity), the dequenching path enables us to predict the extent of conformational arrangement of integrins. Integrin orientation can also be correlated to FRET efficiency using phasor analysis [refer again to Eq. (3)]. An FRET efficiency of 37% was found for the experimental stage, in which ligand-binding titration was performed whereas dequenching of FITC as a result of integrin activation resulted in an efficiency of 26%.

## 5 Discussion

Herein, we correlate changes in fluorescence intensity and fluorescence decay kinetics to changes in integrin conformation, quantifiable by FRET. To acquire FRET measurements, we took mutant-type U937 ΔST lymphoblastic cells, which coexpress VLA-4 integrin dimers and formyl peptide receptor. The activation of the integrins, which was mediated by the formyl peptide receptor, was observed optically and found to occur over a time period that permitted quantification by a flow cytometer that captured total intensity as well as fluorescence lifetime-dependent parameters. These measurements were based on the VLA-4 integrins labeled with LDV-FITC fluorescence donor moieties. With the addition of a red fluorescence acceptor molecule, PKH26, energy transfer was initiated and Förster efficiencies were calculated.

On a qualitative level, the fluorescence intensity changes correspond to our experimental initiation of FRET and loss of FRET. Cells measured before labeling with LDV-FITC express the lowest emission (i.e., autofluorescence), and emission from cells after addition of LDV-FITC (i.e., labeled to the VLA-4 integrins) correspondingly increase to fluorescence intensity levels that are at their brightest. Emission signals from LDV-FITC-labeled cells after addition of the PKH26 acceptor fluorophore are decreased, yet remain above autofluorescence. Likewise, emission reverts to brighter levels after the LDV-FITC- and PKH26-labeled cells are treated with an fMLFF activator (i.e., activating integrins to standing), albeit to levels below the brightest non-FRET stage (i.e., LDV-FITC only). Therefore, expected changes in emission occur when the FRET protocol experiment is performed. Our interpretation of the CV percentages for each cell population (n≈10,000) before, during, and after FRET is that during each stage in the protocol, all cells are labeled uniformly and behaving consistently. When, and if, a greater cell-to-cell difference was found between the FRET stages, the variations are due to the overall lower total donor emission signal detected (i.e., lower signal-to-noise). Intensity-based values are conventional flow cytometric measurements that allow us to quantify the conformational change of the integrin headpiece to a position extending from the cell membrane using the sensitized acceptor emission. Using conventional one-color cytometry, we make the supposition that changes in donor emission, measured across many cells, correlate to differences in the surface integrin states of activation. However, brightness is a function of other factors, such as number of molecules present and quantum yield of each fluorophore. Therefore, our ability to monitor integrins via FRET and using conventional cytometry in this way is limited.

Accordingly, we add TRFC measurements to this study. Our end goal is to (i) measure FRET efficiencies without impact from intensity-based error, (ii) reveal multiple fluorescence lifetimes by the donor, and (iii) estimate the FRET trajectory during this process. With frequency-domain analysis, we are able to collect both phase and fluorescence demodulation lifetimes on a cell-by-cell basis before, during, and after FRET is initiated. Changes in these fluorescence lifetimes were found at time points that correspond to acceptor fluorophore addition and fMLFF activator introduction into the cell suspension. The average phase lifetime of FITC was reduced by 2 ns after the PKH26 was added and then increased by more than a full nanosecond after integrin activation (loss of FRET). Fluorescence lifetime values have small variation cell-to-cell and, thus, outperformed conventional cytometry measurements during our FRET experiment. That is, using fluorescence lifetime as a parameter, we are able to identify cell subpopulations where for example, bimodal lifetime populations are observed for all cells measured during loss of FRET. Such a measurement would imply that during integrin activation a fraction of cells participate in partial-to-no integrin activation. With conventional cytometry measurements such differences would be challenging to decipher.

By measuring the fluorescence lifetime, we can directly identify if FRET occurs and predict FRET efficiency, yet it is also important to realize that FRET acceptor density directly affects FRET efficiency. While the fluorescence lifetime of the donor is not impacted by its concentration, FRET is nonetheless dependent on the acceptor, In prior work on this system, the real-time donor binding kinetics, determination of acceptor density, and spatial distribution of the set of fluorescent probes used in our study were described previously.^9^^,^^43^ Additionally, the FRET efficiency is validated by changes owing to the different vertically extended states of integrins, which cause a range of donor probes’ closest approach to the acceptor probe and extension that is well beyond the proximity of the acceptor probe. This dynamic nature of conformational flexibility creates a heterogeneous receptor population on a given cell’s surface. Yet, as with most other biological systems, despite there being multiple conformations of receptors present, the average receptor conformation is quantitatively observed. Therefore, the mean donor lifetime is dependent on the average conformational state of the integrin that exists. In more detail, if the majority of integrins on the cell are in the resting or “bent” conformation, the donor resides in close proximity to the membrane surface. Where lipid acceptors are incorporated, the donor will exhibit a shorter lifetime. This corresponds to the quenched donor state in the intensity-based FRET assay.^9^ A transition to the extended integrins state that includes a distribution of states (partial and full extension) results in the increase in the mean fluorescence lifetime that corresponds to the unquenching of the donor fluorescence in the intensity-based assays. However, it is still not possible to resolve the confirmation of individual integrins with time-resolved flow cytometric measurements. Our phasor approach represents a step toward quantitative evaluation of heterogeneous clusters in the cell population that exhibit multiple exponential lifetimes. Thus, both single and multiple exponential lifetime analyses are valid, and a distribution of lifetimes indicates a range of different molecular conformations.

With a frequency-domain phasor approach, we are able to make predictions about the behaviors of the cell populations before, during, and after FRET. Furthermore, confidence inferred from phasor graph analysis is substantiated by comparing the average fluorescence lifetime histogram data to the phasor graph data. For events undergoing FRET, histogram analysis showed 31% versus 33% from phasor analysis, while the remainder of events (69% and 67%, respectively) was not undergoing FRET. Loss of FRET histogram data shows 69% events dequenching whereas phasor analysis shows 67% of events dequenching, while 31% and 33% of observed events, respectively, were not responding to the stimuli. Development of a phasor trajectory or “dequenching path” allows us to look more closely at the route the cell populations follow, which in turn leads to the knowledge of FRET efficiency per cell. The overall trajectory is a visual aid for predicting the temporal orientation of integrins as a result of affinity modulation and can be utilized to interpolate the FRET efficiencies at any given point along the calculated trajectory. It is apparent with the phasor plot that multiple fluorescence lifetimes exist, which is expected as multiple integrin conformations can be found per cell, therefore, validating both the single and multiple exponential lifetime analysis presented in this work. The next stage is to take the FRET efficiency values and translate those to an activated integrin concentration using both the loss of FRET path and concentration titration calibration.

## 6 Conclusion

Conformational movement of many integrins is known to be closely tied to their function; therefore, resolving the conformational flexibility of integrins on a truly quantitative level is important to understand the subtleties of how and when integrins activate and the heterogeneity thereof. Herein, experiments with FRET allow us to work toward this goal through the introduction of TRFC and phasor-based analyses. Our cytometric data allow the precise characterization of FRET in order to track integrin conformational changes across large cell populations. With TRFC, data were acquired and used to evaluate levels of activation, and a dequenching trajectory was computed to continuously track the FRET efficiency. With an interpolated trajectory, we are able to calculate the percentages of cells with surface integrins that move from a native resting state, to an intermediate state, and ultimately to a fully activated state. The significance of this is the ability to screen populations of cells against activators that affect integrin activation and thereby quantify if and when no-response, partial response, or full activation occurs.
